# Diagnostic testing for interstitial lung disease in common variable immunodeficiency: a systematic review

**DOI:** 10.3389/fimmu.2023.1190235

**Published:** 2023-05-08

**Authors:** Heba M. Bintalib, Annick van de Ven, Joseph Jacob, Jesper Rømhild Davidsen, Børre Fevang, Leif G. Hanitsch, Marion Malphettes, Joris van Montfrans, Paul J. Maglione, Cinzia Milito, John Routes, Klaus Warnatz, John R. Hurst

**Affiliations:** ^1^ University College London (UCL) Respiratory, University College London, London, United Kingdom; ^2^ Department of Respiratory Care, King Saud bin Abdulaziz University for Health Sciences, Jeddah, Saudi Arabia; ^3^ King Abdullah International Medical Research Centre, Jeddah, Saudi Arabia; ^4^ Departments of Internal Medicine & Allergology, Rheumatology & Clinical Immunology, University Medical Center Groningen, Groningen, Netherlands; ^5^ Satsuma Lab, Centre for Medical Image Computing, University College London (UCL), London, United Kingdom; ^6^ South Danish Center for Interstitial Lung Diseases (SCILS), Department of Respiratory Medicine, Odense University Hospital, Odense, Denmark; ^7^ Odense Respiratory Research Unit (ODIN), Department of Clinical Research, University of Southern Denmark, Odense, Denmark; ^8^ Centre for Rare Disorders, Division of Paediatric and Adolescent Health, Oslo University Hospital, Oslo, Norway; ^9^ Section of Clinical Immunology and Infectious Diseases, Division of Surgery, Inflammatory Medicine and Transplantation, Oslo University Hospital, Oslo, Norway; ^10^ Institute of Medical Immunology, Charité - Universitätsmedizin Berlin, Corporate Member of Freie Universität Berlin and Humboldt Universität zu Berlin, Augustenburger Platz 1 and Berlin Institute of Health, Berlin, Germany; ^11^ Berlin Institute of Health at Charité - Universitätsmedizin Berlin, BIH Center for Regenerative Therapies (BCRT), Charitéplatz 1, Berlin, Germany; ^12^ Department of Clinic Immunopathology, Hôpital Saint-Louis, Paris, France; ^13^ Department of Pediatric Immunology and Infectious Diseases, Wilhelmina Childrens Hospital, University Medical Center Utrecht (UMC), Utrecht, Netherlands; ^14^ Section of Pulmonary, Allergy, Sleep, and Critical Care Medicine, Chobanian & Avedisian School of Medicine, Boston University, Boston, MA, United States; ^15^ Department of Molecular Medicine, Sapienza University of Rome, Rome, Italy; ^16^ Division of Allergy, Asthma and Immunology, Department of Pediatrics, Medicine, Microbiology and Immunology, Medical College Wisconsin, Milwaukee, WI, United States; ^17^ Department of Rheumatology and Clinical Immunology, Medical Center - University of Freiburg, Faculty of Medicine, University of Freiburg, Freiburg, Germany; ^18^ Center for Chronic Immunodeficiency (CCI), Medical Center - University of Freiburg, Faculty of Medicine, University of Freiburg, Freiburg, Germany

**Keywords:** CVID, interstitial lung disease, GLILD, diagnosis, systematic review

## Abstract

**Introduction:**

Common variable immunodeficiency related interstitial lung disease (CVID-ILD, also referred to as GLILD) is generally considered a manifestation of systemic immune dysregulation occurring in up to 20% of people with CVID. There is a lack of evidence-based guidelines for the diagnosis and management of CVID-ILD.

**Aim:**

To systematically review use of diagnostic tests for assessing patients with CVID for possible ILD, and to evaluate their utility and risks.

**Methods:**

EMBASE, MEDLINE, PubMed and Cochrane databases were searched. Papers reporting information on the diagnosis of ILD in patients with CVID were included.

**Results:**

58 studies were included. Radiology was the investigation modality most commonly used. HRCT was the most reported test, as abnormal radiology often first raised suspicion of CVID-ILD. Lung biopsy was used in 42 (72%) of studies, and surgical lung biopsy had more conclusive results compared to trans-bronchial biopsy (TBB). Analysis of broncho-alveolar lavage was reported in 24 (41%) studies, primarily to exclude infection. Pulmonary function tests, most commonly gas transfer, were widely used. However, results varied from normal to severely impaired, typically with a restrictive pattern and reduced gas transfer.

**Conclusion:**

Consensus diagnostic criteria are urgently required to support accurate assessment and monitoring in CVID-ILD. ESID and the ERS e-GLILDnet CRC have initiated a diagnostic and management guideline through international collaboration.

**Systematic review registration:**

https://www.crd.york.ac.uk/prospero/, identifier CRD42022276337.

## Introduction

Common variable immunodeficiency disorders (CVID) are the most prevalent primary symptomatic immunodeficiencies (PID), characterised by hypogammaglobulinemia and impaired immune responses to infections and vaccinations (1, 2). The two major clinical manifestations of CVID are recurrent, mainly bacterial infections and complications secondary to dysregulation of the immune system. Infections can be largely prevented through appropriate use of intravenous or subcutaneous immunoglobulin replacement therapy (IgRT) (3, 4). However, non-infectious complications such as interstitial lung disease, cytopenias, gastrointestinal and hepatic disease, and lymphoproliferative disease are difficult to manage and have become the major causes of morbidity and mortality (4).

Ten to 20% of people with CVID develop CVID-associated interstitial lung disease (CVID-ILD), histologically characterised by granulomatous inflammation and/or lymphocytic infiltrates (5). The condition has also been termed granulomatous and lymphocytic interstitial lung disease (GLILD). CVID-ILD appears alongside other non-infectious complications that increase morbidity and mortality in this group of patients, and thus is considered a manifestation of systemic lymphoproliferation and immune dysregulation (5, 6). There is no single clinical finding or investigation that facilitates the diagnosis of CVID-ILD due to heterogeneity of the disease. CVID-ILDs share clinical and histological characteristics with other conditions, and there is currently no single consensus on the diagnostic criteria for CVID-ILD. The understanding of pathogenesis is limited, and significant gaps in knowledge about diagnosis and management remain (5, 7). No evidence-based guideline for diagnosis or treatment is currently available, and management generally relies on clinicians’ expert opinions (7, 8).

The aim of this systematic review is to provide a comprehensive overview on diagnostic tests employed by clinicians when assessing adult and paediatric patients with CVID for possible CVID-ILD, reporting the utility and risks of these tests, and highlighting tests informing on disease activity or progression.

## Method

We searched Ovid-EMBASE, MEDLINE, CINAHL PLUS and PubMed to identify all relevant published articles using the following key words: common variable immunodeficiency, late onset hypogammaglobulinemia, interstitial lung disease, lymphocytic interstitial pneumonitis, granulomatous lymphocytic interstitial lung disease, diagnosis, sign, symptom, clinical feature, characteristic, and manifestation.

Our inclusion criteria were: (1) type of study: we included prospective and retrospective cohort studies, case control studies, case reports, case series and non-randomised controlled trials. (2) population: individuals who fulfilled clinical criteria for common variable immunodeficiency, with or without genetic underlying diagnosis and confirmed or suspected ILD. (3) studies that reported information on diagnostic testing for ILD in patients with CVID. (4) outcomes: utility and, where reported, risks of diagnostic tests. (5) studies were in English. We excluded abstracts, theses, book chapters, review articles, and opinion articles, but searched the reference lists of reviews for primary sources. The original search was done on June 15, 2022, and was updated to December 2^nd^, 2022. The protocol was registered on PROSPERO (registration: CRD42022276337).

Studies retrieved using the search strategy were entered into Rayyan software (https://www.rayyan.ai/). All titles and abstracts were assessed by two reviewers against the inclusion criteria. Conflicts were settled by a third reviewer. Reviewers read the entire paper if the title and abstract didn’t provide enough information. The references were examined for additional sources. The primary data collected included study design, characteristics of study participants (where reported), description of the diagnostic method, test characteristics, and an evaluation of diagnostic utility. The results were collated for narrative synthesis. Only qualitative data were synthesised.

### Qualitative assessment of study methodology

The assessment of study quality was completed by one author. To evaluate bias among observational studies, we used the Newcastle-Ottawa Scale (NOS) and a modified NOS which assesses studies based on three broad perspectives: the selection of the study groups; the comparability of the groups; and the ascertainment of either the exposure or outcome of interest (for cohort, case-control, or cross sectional studies respectively) (9). The Joanna Briggs Institute (JBI) Critical Appraisal Tools was used for case reports and case series (10). This addresses the risk of bias and internal validity and comprises 10 questions about confounding, selection (bias), information bias, and clear reporting.

## Results

**Figure 1:**
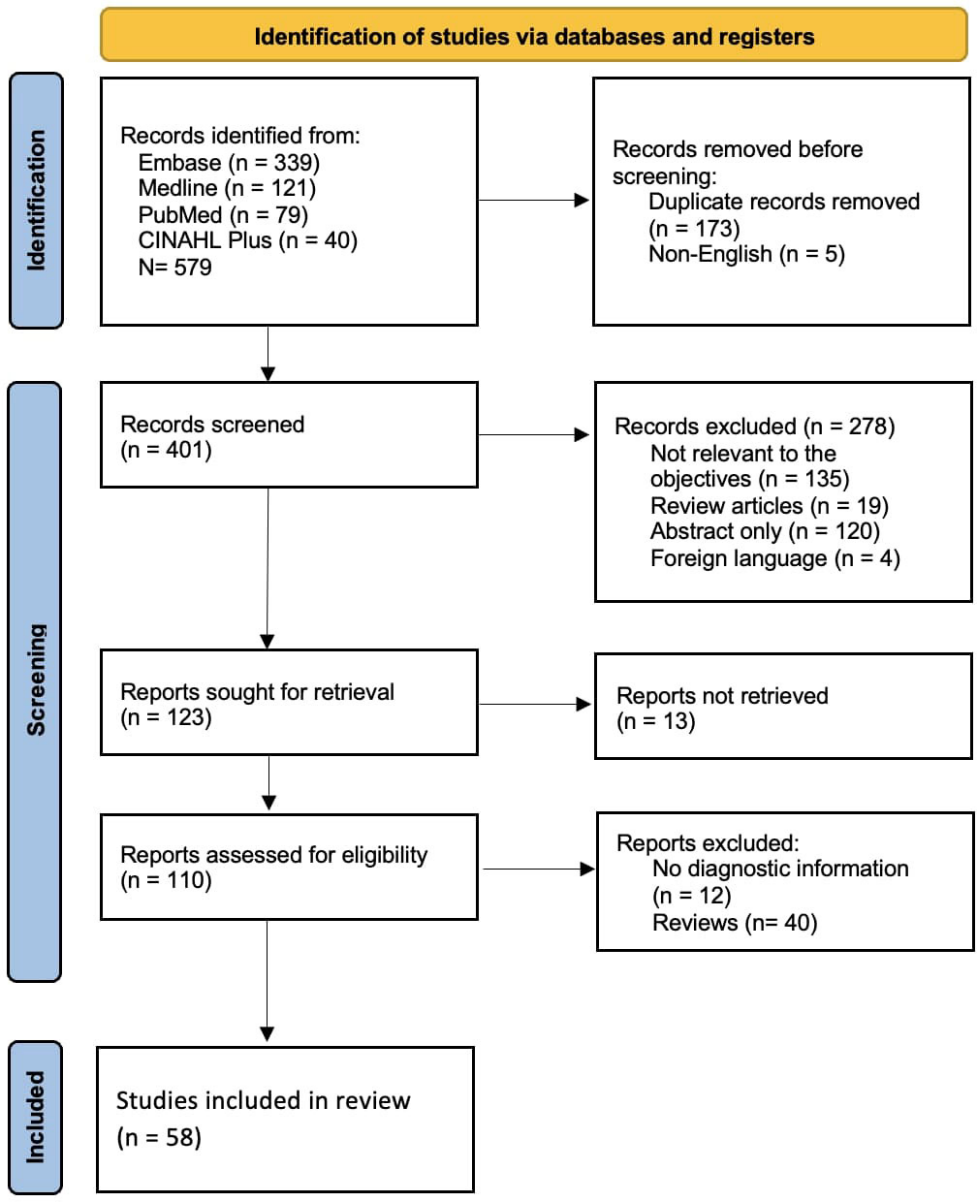
PRISMA flow diagram of study selection process. *From*: (11). For more information, visit: http://www.prisma-statement.org/.

In total 58 studies describing a total of 796 patients were included (**Figure 1**). The average age at diagnosis of CVID-ILD in 422 adult and 28 paediatric patients was 40 years and 11 years, respectively. Not all papers were primarily aiming to evaluate diagnostic tests, but all papers that met our inclusion criteria were included. Thirty (52%) studies were performed in Europe, 24 (41%) were in the United States, and the remaining 4 (7%) were in Japan, Australia and Argentina. Among the 58 articles, 40 referred to the condition as GLILD, 16 studies used CVID related ILD and 2 studies described the condition as granulomatous CVID. We will use CVID-ILD in this review.

The designs of the studies we included, and population characteristics of people included in these studies are reported in **Table 1**. Results summarising the frequency of the use of the diagnostic tests and the prevalence of abnormalities detected are reported in **Table 2**. Since most of the included studies involved an observational design, we considered the overall quality of evidence to be low. **Supplementary Tables 1**–**5** provide a summary of the quality assessment.

**Table 1:** Study design and population characteristics.

Characteristics	Studies *n* = 58	%
Design		
Case reports	21	36
Case series	11	19
Cohort	17	29
Case control	5	9
Cross sectional	4	7
Age group		
Adults only	48	83
Children (age < 18 years)	8	14
All ages	2	4

**Table 2:** Diagnostic tests in the evaluation of suspected CVID-ILD.*

Test	n = number of studies (%)	Testing for diagnostic evaluation	Positive test required for diagnosis	Number of Patients *n=*	
				Abnormal	Normal
**Radiology tests**		57	57		
Chest X-ray	16 (29)			40	0
CT chest	58 (100)			675	0
PET	6 (11)			38	0
**Bronchoalveolar lavage**		24	2		
Virology or microbiology	17 (30)			17	52
Differential cell count	15 (27)			96	29
**Pulmonary function tests**		40			
Spirometry	40 (73)			76	29
DLCO	25 (45)			68	13
**Biopsy**		42	42		
TBB	17 (30)			29	28
TBLC	1 (2)			1	0
VATS	17 (30)			81	0
Thoracotomy (open surgery)	7 (13)			11	0
Lung but not specified	13 (23)			170	NC
Other site	7 (30)			57	
**Blood work-up**	35 (61)	23		†	
**Genetic**	17 (33)	17		48	52

CT, computed tomography; PET, positron emission tomography; TBB, Transbronchial biopsy; TBLC, Transbronchial lung cryobiopsy; VATS, Video-Assisted Thoracic Surgery; DLCO, diffusion of the lungs for carbon monoxide; NC, not clear. * References of the studies where these numbers refer to are in the **Supplementary Table S6**. † See text for details.

### Radiology

Abnormal lung imaging is considered a prerequisite for the diagnosis of CVID-ILD. Radiology studies were therefore the most frequently used tool for the assessment of potential lung involvement. Chest radiographs (CXR) were reported in 16 articles (5, 12–26). Typical pulmonary findings were bilateral patchy and nodular opacities with lower lung field predominance. In all of these studies (high-resolution/thin-section) Computed Tomography (CT) was subsequently performed because plain radiographs were not considered diagnostic.

**Figure 2:**
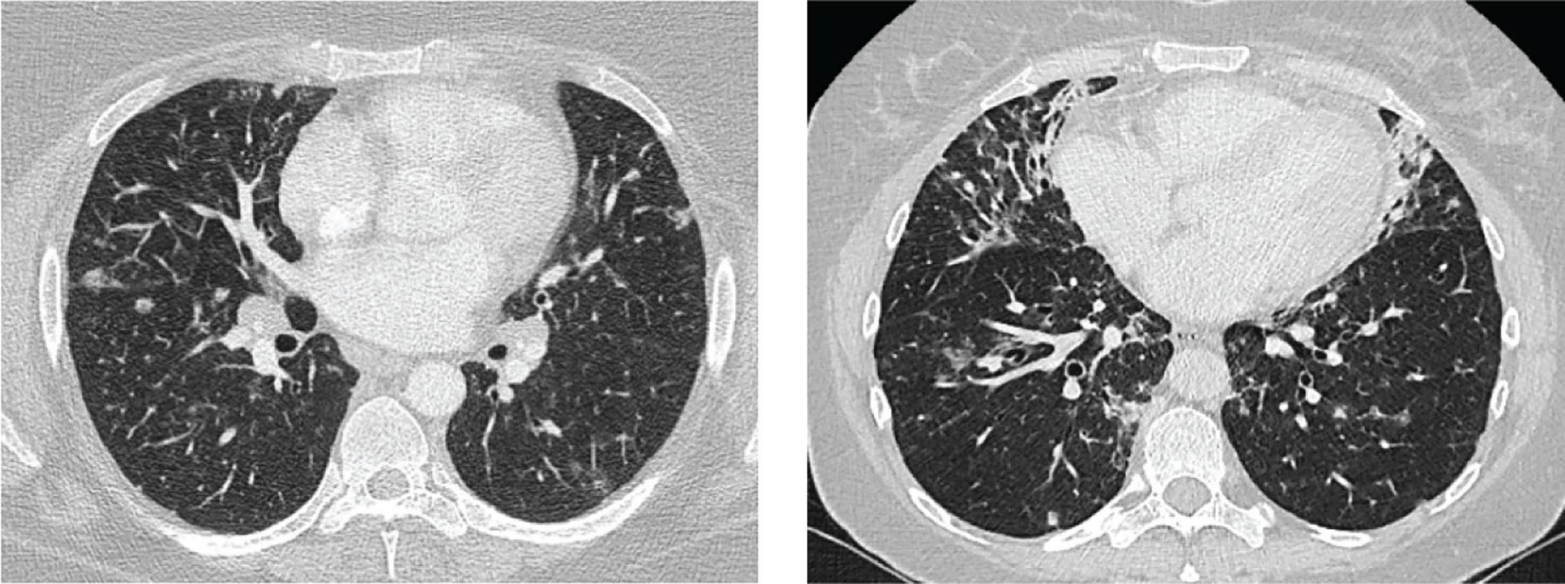
Images of two patients. Left: diffuse nodules and lymphadenopathy. Right: combination of diffuse nodules, reticulation and ground-glass opacities. Apart from CVID-ILD features, there are also signs of airway disease. From reference (51) with permission.

Thirty-one case reports and case series reported the use of CT in the diagnostic work up (12–22, 26–45). Thirteen observational studies relied primarily on CT as their only criteria for CVID-ILD (2, 23, 27, 46–55), while 13 studies required either histological confirmation (5, 24, 25, 56–63) or an impairment in pulmonary function (64, 65) in addition to the detection of relevant CT abnormalities. As CT imaging was the basis for the diagnosis of CVID-ILD, CT imaging was abnormal in all patients. Studies which relied on CT-features alone to make the diagnosis of CVID-ILD defined typical pulmonary findings as: the presence of micronodules (which were predominantly peri-bronchovascular and more frequently found in the lower lobes), ground glass opacities, consolidation and interlobular septal thickening (**Figure 2**). In addition, thoracoabdominal lymphadenopathy and splenomegaly were characteristic extrapulmonary features. One recent paper by Smits et al. was added to the review despite being published after our search was completed, as it provides additional insight into the diagnostic criteria used for CVID-ILD (55). The authors recruited patients from the STILPAD study in which appropriate radiographic signs of CVID-ILD were sufficient for diagnosis. According to the authors’ classification, a ‘possible’ diagnosis was made if patients presented radiographic signs of CVID-ILD only, whereas a ‘probable’ diagnosis required either a probability score >50% and radiographic signs of CVID-ILD or histological confirmation of CVID-ILD (55).

Seven studies reported using positron emission tomography-computed tomography (PET-CT) for assessing possible CVID-ILD (20, 30, 34, 35, 37, 39), assessing disease activity (50), and/or monitoring the response to treatment (30). In a retrospective cohort of 32 patients with CVID-ILD, Fraz et al. found that patients with progressive disease based on lung function tests had significantly higher mean standardized uptake values (SUV) in their lungs at baseline. This suggests a potential role of PET-CT in detecting pulmonary inflammation as part of active or uncontrolled overall disease (50).

The use of Magnetic Resonance Imaging (MRI) was not commonly reported as a diagnostic test for CVID-ILD. However, a few studies suggest that MRI scanning can be used an alternative to CT scanning to detect lung alterations and reduce radiation exposure in people with primary immune deficiencies (66–68).

### Pulmonary function tests

Pulmonary function tests (PFT) as assessment tools were reported in 40 studies (5, 13, 15–18, 20–24, 27, 28, 30–36, 38–45, 49, 52–54, 56, 57, 59, 60, 62–64, 69), and included spirometry, measurement of diffusion capacity and assessment of static lung volumes (i.e., total lung capacity (TLC), and residual volume (RV)). Two studies reported PFT abnormalities as potential diagnostic criteria for CVID-ILD in addition to CT (64, 65). In 105 CVID-ILD patients with reported results, 53 (50%) patients had a restrictive lung pattern, while 20 (19%), 29 (28%) and 3 (3%) had obstructive, normal and mixed results, respectively. Gas transfer was low in 68 (57%) patients.

### Bronchoalveolar lavage

Bronchoalveolar lavage (BAL) was generally performed to exclude infections, including bacteria, Mycobacteria, fungi and respiratory viruses. Seventeen studies reported BAL culture to exclude infection (12, 13, 16–19, 21, 22, 26, 31, 32, 35, 37, 41, 42, 44, 65) but only four reported polymerase chain reaction (PCR) to exclude cytomegalovirus, Epstein-Barr virus (EBV), HIV and Mycoplasma pneumonia (13, 35, 37, 41). The most common respiratory pathogens reported, where a pathogen was detected, were *Staphylococcus aureus*, *Haemophilus influenzae*, *Streptococcus pneumoniae*, rhinovirus, and cytomegalovirus.

Flow-cytometry analysis including differential cell count was reported in 15 studies verifying significant lymphocytosis in 96/125 (78%) of patients (15–17, 22, 24, 26, 35, 42, 44, 57–59, 64, 65, 69). In addition, where lymphocyte phenotyping was performed this showed a larger proportion of B cells, predominantly CD21low B cells (57, 65). Friedmann et al. reported that patients with CVID-ILD had fewer regulatory T cells, but more T follicular helper (TFH)-like memory cells skewed towards Th1 cells, as well as a greater proportion of B cells (particularly the inflammatory CD21low B cell subtype) in BAL compared to sarcoidosis (65). There are conflicting reports regarding CD4/CD8 ratios in BAL, which have been described as reduced, elevated, and normal (24, 58, 59, 65).

### Biopsy

The diagnosis of CVID-ILD was confirmed by biopsy in 31 case reports and series (12–22, 26–45) and was used as an obligatory inclusion criterium for CVID-ILD patients in eleven studies (5, 24, 25, 54, 56–60, 62, 63). Transbronchial biopsy (TBB) was described in 17 studies involving 57 patients, 28 of whom had definitive results, while the remaining patients underwent a supplemental biopsy modality to confirm the diagnosis (16–18, 22, 30, 31, 34, 38, 42, 44, 50, 58, 59, 62–64, 69). The most common diagnostic findings were non-necrotizing granulomatous and lymphocytic inflammation. The use of video-assisted thoracoscopic surgery (VATS) was reported in 17 studies (15, 16, 21, 26, 33, 34, 36, 39–41, 45, 47, 57, 60, 62, 63, 69). The results from 81 patients demonstrated the characteristic histological findings of CVID-ILD. Ten studies reported the use of open biopsy in 11 patients where all had conclusive results (12–14, 18, 28, 29, 42, 60, 63). The most common findings on surgical biopsy were non-necrotising granulomatous inflammation, lymphoid interstitial pneumonitis (LIP), and/or lymphoid hyperplasia, while organising pneumonia (OP), interstitial fibrosis and follicular bronchiolitis were less common. Only one article reported the use of transbronchial cryobiopsy (TBCB), in one patient with no conclusive results (44). One case report used transbronchial fine-needle aspirate (FNA) of pulmonary nodules to exclude malignancy and lymphoma (20). Extrapulmonary biopsy was accepted to substantiate the diagnosis of CVID-ILD in seven studies (24, 28, 32, 37, 42, 54, 57). Lymph node biopsy was the most frequently reported, followed by liver, spleen and skin. The most common finding was non-necrotizing granulomata.

Only one study reported the risk of biopsy-related complications, in this case related to the VATS procedure, where the patient developed pleural empyema (26). Biopsy samples were often also tested for fungi, mycobacteria, pneumonia, EBV, and CMV using culture, special stains and molecular biology.

### Blood biomarkers and genetic testing

The blood work-up differed markedly between studies. As a result, drawing conclusions was challenging because no one blood biomarker is has been shown to aid the diagnosis of CVID-ILD. Fraz et al. recently reported that CVID-ILD patients have elevated serum markers of T cell activation and exhaustion reflected by elevated level of TNF, IFN-γ, sCD25, and sTIM-3; increased concentrations of pulmonary epithelium injury biomarkers including CC16, SP-D and MMP-7; and increased levels of ECM remodelling markers compared to patients with other non-infectious complications. Other potential biomarkers have been used to developed diagnostic prediction models and to help avoid biopsy (as discussed further below). Furthermore, different blood biomarkers have been reported to be associated with CVID-ILD progression, and these include increased level of B cell-activating factor (BAFF), IgM in serum, the soluble form of the interleukin-2 receptor (sIL-2R) and neopterin (17, 48, 55, 70). These data are summarised in **Table 3**. Smits et al. have reported that neopterin levels, in addition to IgM level and sIL-2R, may have the potential to serve as biomarkers for disease activity (55).

**Table 3:** Studies that evaluated biomarkers of CVID-ILD disease activity and progression.

Author/year	Study design	Aims	Treatments administered	Outcome Predicted	Follow-up time	Indicators Examined	Outcomes
Vital et al., 2015 (17)	Case report	To highlight the clinical improvement observed in the patient after the initiation of combination therapy and to report the potential of serum levels of IL-12 and soluble IL-2 receptor (sil-2R) to use as disease biomarker.	Rituximab at a dose of 375 mg/M2 weekly for four weeks and repeated every 6 months for a total of 3 courses and oral azathioprine (1.7 mg/kg/d) to complete a total of 18 months.	Disease activity	4 months	CT, PFT, and serum levels of IL-12 and sIL-2R.	Improved CT and PFT. Normalize level of serum IL-12, sIL-2R, ACE, and erythrocyte sedimentation rate level. *Serum IL-12 and sil-2R may hold some promise as clinically useful biomarkers of disease activity and/or response to treatment in GLILD
Jolles et al., 2016 (30)	Case report	To describe the use of combined 2-[ (18)F]-fluoro-2-deoxy-d-glucose positron emission tomography and computed tomography (FDG PET-CT) scanning for the assessment and monitoring of response to treatment in a CVID-ILD patients.	Two doses of (1 g) of rituximab and mycophenolate mofetil.	Disease activity	2 months	Clinical, PFT, and FDGPET-CT scan.	- Improved FVC and DLCO. - FDG PET-CT imaging detected a reduction metabolic activity in abnormal tissue after treatment. * FDG PET-CT imaging detected high metabolic activity in abnormal tissue that may respond to treatment
Maglione et al., 2015 (47)	Cohort	To determine if all CVID with ILD have physiological worsening, and if clinical and/or laboratory parameters may correlate with disease progression.	*	Disease progression	20 or more months.	Clinical, PFT, and serum immunoglobulins.	Progressive CVID-ILD was significantly related to reductions in FEV1, FVC, DLCO, lower mean IgG levels, and an increase in levels of IgM, with more significant thrombocytopenia.
Maglione et al., 2019 (48)	Cohort	Interrogated the clinical and laboratory parameters aiming to identify a biomarker that distinguishes those with ILD progression.	*	Disease progression	18 months.	Blood and lung samples using culture, cytometry, ELISA, and histology.	Increase level of serum IgM and increased B cell–activating factor (BAFF) significantly related to ILD progression.
Fraz et al., 2020 (50)	Cohort	To compare clinical, immunological, and radiological (including both CT and FDG PET/CT) features in patients with stable or progressive GLILD based on functional pulmonary testing.	Nine patients received 1 g rituximab intravenously 2 weeks apart, every 6 months. 2 patients received it as monotherapy. 7 patients combined it with 100–200 mg azathioprine.	Compare between stable and progressive	Median follow-up time was 123 months.	PFT, CT, immunoglobulin levels and T- and B-cell subpopulations, and FDG PET/CT.	Progressive GLILD were defined as - had an absolute decline in FVC percent predicted > 10 percentage points (p.p.) And/or DLCO percent predicted >15 p.p. - had significantly greater pathology on pulmonary CT - had significantly higher mean standardized uptake value (SUVmean), metabolic lung volume (MLV) and total lung glycolysis (TLG) as compared to patients with stable disease. Rituximab was followed by a significant improvement in overall pulmonary CT pathology, while changes in pulmonary function varied.
Van Stigt et al., 2021 (70)	Case-control	To determine whether serum sil-2R level can be used as a low invasive biomarker for detection of granulomatous disease and for monitoring granuloma progression or remission in CVID patients.	Three patients received Rituximab; one patient received prednisone in combination with methotrexate.	Disease progression	6 months.	Serum sil-2R measurements	- Sil-2R levels rise with progression of granulomatous disease and decline upon remission. - Decrease in sIL-2R levels was observed after the treatment.

* No evaluation of treatment response.

Genetic evaluations were reported in seventeen studies (23, 25, 28, 32, 39, 41, 43–45, 48, 50, 56, 57, 62–64, 69). 48 of 100 reported patients had cytotoxic T lymphocyte antigen 4 (CTLA-4) haploinsufficiency, or transmembrane activator and calcium-modulating cyclophilin ligand interactor (TACI) (TNFRSF13B) or signal transducer and activator of transcription 3 (STAT-3) mutations.

### Diagnostic prediction models

Four studies developed prediction models for biopsy-positive CVID-ILD based on clinical, laboratory and/or lung physiological parameters to assist predicting the presence of CVID-ILD (54, 56, 57, 59). These are reported in **Table 4**. All studies reported splenomegaly as a predictor for CVID-ILD, with odds ratios between 8.47 and 23.9 (54, 56, 57, 59). Cinetto et al. proposed a CVID-ILD predictive model based on splenomegaly, CD21lo B cells percentage, autoimmune cytopenia and DLCO percent predicted with Area Under the Curve (AUC) of 0.98 (57). The recent predictive model proposed by Cabanero et al. was based on splenomegaly, lymphadenopathy, low CD8 cell in BAL, and high Baumann’s CVID-ILD composite score, with an AUC of 0.985 (56). Such studies need external validation.

**Table 4:** Prediction models to screen patients with CVID-ILD.

Author/Year	Country	Study design	Control n=	CVID-ILD n=	CVID-ILD diagnosis based on	Predictors	OR	95% CI	AUC
Mannina et al., 2016 (59)	USA	Case-control	52	34	HRCT and biopsy	Hypersplenism	23.9	4.5–179.10	0.92
						Polyarthritis	18.7	2.3–206.86	
						FVC less than 80% predicted	0.93	0.87–0.98	
Hartono et al., 2017 (54)	USA	Case-control	26	26	HRCT, biopsy, and BAL	Splenomegaly	17.3	3.9-74.5	0.86
						ITP or AIHA	4.8	1.1-20.2	
						Low serum IgA level (<13 mg/dl)	3.6	1.2-11.9	
						Percentage of CD21low B cells >5%	5.8	1.6-24.7	
Cinetto et al., 2021 (57)	Italy	Cross-sectional	125	47	HRCT, biopsy, and BAL	Splenomegaly	8.47	1.06-67.20	0.98
						Autoimmune cytopenia	45.17	4.76-428.56	
						CD21low B cells percentage	1.2	1.06-1.36	
						DLCO percent predicted	0.94	0.89-0.99	
Cabanero et al., 2022 (56)	Spain	Cross-sectional	50	7	HRCT, biopsy, and BAL	Splenomegaly	9.42		0.985
						Lymphadenopathy	6.25		
						Low CD8 cell in BAL	0.9		
						High Baumann’s CVID-ILD composite score	1.56		

OR, odd ratio; CI, confidence interval; AUC, area under the ROC curve; HRCT, high-resolution Computed Tomography; BAL, bronchoalveolar lavage; ITP, immune thrombocytopenia; AIHA, autoimmune hemolytic anemia.

## Discussion

Managing clinically relevant complications in a rare disease is a significant challenge for clinicians, especially in the absence of evidence-based guidelines. The diagnosis and managing of CVID-ILD therefore usually depends on the decisions and experience of individual clinical teams. In this systematic review we reviewed diagnostic methods and criteria for CVID-ILD, and for informing prognosis in CVID-ILD. The key findings are (i) in general, there was diagnostic consistency across studies, (ii) HRCT was the most frequently reported test to detect CVID-ILD, (iii) lung biopsy is required to definitively confirm the diagnosis but some teams make a clinical diagnosis, (iv) BAL was routinely performed to exclude infection, and (v) non-biopsy prediction models for CVID-ILD had good discriminative accuracy but require external validation. A more consistent diagnostic approach would facilitate research collaboration and comparisons across studies (8).

The term GLILD was introduced by Bates et al. to describe a group of CVID patients with histological findings of LIP, lymphoid hyperplasia, follicular bronchiolitis, and/or granulomatous disease (5). However, the term has been interpreted differently across the literature. Some authors consider pulmonary fibrosis and organising pneumonia (OP) as additional features, while others consider the diagnosis to require histologically proven pulmonary granuloma. Thus, there is discussion to reconsider terminology (46, 71–73). We found that three-quarters of the included papers refer to these (histological) manifestations as GLILD, but prefer the term CVID-ILD.

The diagnosis of CVID-ILD has been clearly described in case reports and series. In contrast, the inclusion criteria in observational studies completed for other reasons were often vaguely described, which made it challenging to interpret the results. We found general consistency in the diagnostic approach between studies. However, not all tests were always performed in all subjects, notably biopsy.

Patients with CVID-ILD often have other lymphoproliferative and autoimmune manifestations. Splenomegaly, lymphoproliferative disorders, cytopenias such as thrombocytopenia and autoimmune haemolytic anaemia (AIHA) are the most common extrapulmonary manifestations in these patients. The presence of these features could increase the suspicion of CVID-ILD and were used along with other clinical and laboratory features to develop prediction models (as described above). The purpose of these models was to support the diagnosis of CVID-ILD and/or the risk of future CVID-ILD; however, they need to be validated.

Radiology was the investigation most commonly used during the process of diagnosis. HRCT was the most frequently reported test, in all the included articles, as abnormal results usually first raise suspicion of ILD in CVID. Since plain radiographic studies have low sensitivity to provide sufficient diagnostic information, the diagnosis was generally based on abnormalities revealed on CT scan. Few studies employed CT scoring methods to evaluate lung involvement and progression, which can be complex and therefore time-consuming. Meerburg and colleagues evaluated the Baumann and Hartmann scoring methods in a cohort of 138 people with CVID-ILD (51). They reported Hartmann’s scoring to be more reproducible than Baumann’s and suggested use of radiological scoring to measure outcomes in future studies. Despite widespread use of CT, the potential risk of radiosensitivity in CVID should be considered (74, 75) given that scans may need to be repeated over time. MRI can be utilized to evaluate the lungs in patients with CVID as it is comparable to HRCT in its ability to identify bronchial and parenchymal abnormalities (67, 68). Additionally, MRI does not pose the risk of ionizing radiation, which may be a concern for some patients. However, it is important to note that the spatial resolution of MRI may be lower than that of HRCT, which may limit its sensitivity for detecting some lung abnormalities particularly small nodules.

Lung biopsy was the second most common reported test required for diagnosis, and surgical lung biopsy (SLB) had more conclusive results compared to TBB alongside histological diagnosis of other ILDs (76). Histological findings may be diverse between patients, and indeed diverse between different areas of the lung in individual patients. This variability has implications for the amount of tissue collected during sampling. As the volume of sampling increases, the probability of discovering additional features, if not all, of what is referred to as CVID-ILD increases. Verbsky et al., in their longitudinal retrospective analysis evaluating treatments response, reported 34/39 patients had VATS with sampling of at least two areas of the lung in which all patients exhibited at least three of the four main histological abnormalities considered characteristic of CVID-ILD. This could explain why almost half of the patients who underwent TBB had inconclusive results as the small amount of tissue attributed obtained can result in sampling error and thus may not represent the complete histopathological pattern. In addition, the timing of samples with regard to natural history of the disease (or previous administration of medicines) could contribute to the heterogeneity of histological findings (71). When taking complications into account, other diagnostic histological approaches such as TBCB should be also considered for CVID-ILD (76, 77). Currently, histology is regarded necessary for definitive confirmation because it is still unclear how accurately clinic, laboratory, CT and potentially BAL parameters can exclude alternative diagnoses. Consequently, it has been suggested that a classification of probable vs biopsy-proven CVID-ILD is introduced as used with clinical, radiological and histological classification of other ILD subtypes (55, 78, 79). Three of four non-biopsy prediction models for CVID-ILD had good discriminative accuracy in their development studies (56, 57, 59).

Analysis of BAL was often conducted, primarily to exclude infection, although the BAL differential cell count has been described as an adjunct to positive diagnosis of CVID-ILD. 78% of patients had an increased proportion of lymphocytes which was described as the most prominent feature of BAL with expansion of both T-cells and B-cells, predominantly CD21low B cells, which has been utilized as a predictive parameter in two studies mentioned above. This is in contrast with sarcoidosis where there is no increase in B cells, however a diagnosis of sarcoid instead of CVID-ILD can be more readily clarified by simple measurement of serum immunoglobulins (65).

Our results demonstrate that PFTs including gas transfer are widely used during the diagnostic process. However, results vary from normal to severely impaired, in the latter case typically with a restrictive pattern and reduced gas transfer. PFTs abnormalities can often be found but are not sufficiently sensitive to diagnose CVID-ILD. Gas transfer abnormalities are the most common findings. Future studies need to evaluate how valuable PFTs including DLCO are in determining the need for treatment and to assess changes at follow up. Paediatric articles reported less use of PFTs due to challenges conducting the tests in very young children.

This is the first systematic review to evaluate diagnostic approaches in CVID-ILD. There were some limitations of this study. First, we recognise the heterogeneity of the definition and terminology of ILD in CVID, and methodologies used between studies. Second, we could not summarise risks and benefits of the different diagnostic procedures as these were often not reported. Third, the quality of the evidence is generally low, being based on case reports and case series. Finally, we limited our search to include only English articles. A strength of this review is that we collated all evidence in regard to the clinical approach to diagnosis of CVID-ILD by including case reports and series in our evaluation.

Patients with CVID who experience respiratory symptoms, have abnormal imaging findings, or demonstrate decreased lung function should be evaluated for ILD. The risk of CVID-ILD may increase in patients with other autoimmune conditions. Thus, multidisciplinary discussion is crucial in the diagnosis and management of CVID-ILD, as it facilitates a comprehensive and tailored approach to care that can lead to better outcomes and improved quality of life for patients. In addition, consensus diagnostic criteria are urgently required to support accurate assessment and monitoring in CVID-ILD. The European Society for Immunodeficiencies (ESID) has initiated production of a diagnostic and management guideline through international collaboration. The guideline will promote collaboration and disease management, and reduce unwarranted variation in care.

## Data availability statement

The original contributions presented in the study are included in the article/**Supplementary Material**. Further inquiries can be directed to the corresponding author.

## Author contributions

JH selected the review’s subject and directed the research and writing processes. HB, JH, and KW created the search strategy. HB and JH review papers for inclusion and created the tables. JH and KW gave advice during the synthesis of the results. HB wrote the initial draft. AV, JJ, JD, BF, LH, MM, JM, PJM, CM, JR, KW, and JH evaluated and commented on the draft papers. All authors participated to and approved the final draft of the article.
