# Supplementary material for: Diagnostic testing for interstitial lung disease in common variable immunodeficiency: a systematic review
Source: Front Immunol. 2023 May 8;14:1190235. doi: 10.3389/fimmu.2023.1190235 (PMC10200864; doi:10.3389/fimmu.2023.1190235)
Supplement: Supplementary file 1 [file DataSheet_1.docx]

**Supplementary Appendix Content**

[Table S1: JBI Critical Appraisal Checklist for Case Reports. 2](#_Toc129781995)

[Table S2: JBI Critical Appraisal Checklist for Case series. 4](#_Toc129781996)

[Table S3: Quality of included cohort studies (Newcastle-Ottawa Scale, NOS). 5](#_Toc129781997)

[Table S4: Quality of included case-control studies (NOS). 6](#_Toc129781998)

[Table S5: Quality of included cross sectional studies (NOS- adapted for cross sectional studies). 7](#_Toc129781999)

[Table S6: References for table 2 Diagnostic tests in the work-up of suspected GLILD. 8](#_Toc129782000)

# Table S1: JBI Critical Appraisal Checklist for Case Reports.

| **Checklist** | **Sacco et al. 1996** | **Davies et al. 2000** | **Thatayatikom et al. 2005** | **Matsubara et al. 2008** | **Boujaoude et al. 2013** | **Vitale et al. 2015** | **Jolles et al. 2016** | **Tashtoush et al. 2016** | **Pathria et al. 2016** | **Hasegawa et al. 2017** |
| --- | --- | --- | --- | --- | --- | --- | --- | --- | --- | --- |
| 1. Were patient’s demographic characteristics clearly described? | Yes | Yes | Yes | Yes | Yes | Yes | Yes | Yes | Yes | Yes |
| 2. Was the patient’s history clearly described and presented as a timeline? | Yes | Yes | No | Yes | no | Yes | Yes | Yes | Yes | Yes |
| 3. Was the current clinical condition of the patient on presentation clearly described? | Yes | Yes | Yes | Yes | Yes | Yes | Yes | Yes | Yes | Yes |
| 4. Were diagnostic tests or assessment methods and the results clearly described? | Yes | Yes | Yes | Yes | Yes | Yes | Yes | Yes | Yes | Yes |
| 5. Was the intervention(s) or treatment procedure(s) clearly described? | Yes | Yes | Yes | Yes | Yes | Yes | Yes | Yes | Yes | Yes |
| 6. Was the post-intervention clinical condition clearly described? | Yes | Yes | Yes | Yes | Yes | Yes | Yes | Yes | Yes | Yes |
| 7. Were adverse events (harms) or unanticipated events identified and described? | Yes | Yes | No | Yes | NA | NA | NA | NA | NA | Yes |
| 8. Does the case report provide takeaway lessons? | Yes | Yes | Yes | Yes | Yes | Yes | Yes | Yes | Yes | Yes |
| **Total score** | 8 | 8 | 6 | 8 | 6 | 7 | 7 | 7 | 7 | 8 |

UC, unclear; NA, not applicable.

**Table S1: Continued.**

| **Checklist** | **Zdziarski et al. 2017** | **Routes et al. 2017** | **Arraya et al. 2018** | **Shah et al. 2018** | **Sood et al. 2018** | **Tillman et al. 2019** | **Tessarin et al. 2019** | **Cowen et al. 2020** | **Pattanaik et al. 2021** | **Nishimura et al. 2022** |
| --- | --- | --- | --- | --- | --- | --- | --- | --- | --- | --- |
| 1. Were patient’s demographic characteristics clearly described? | Yes | Yes | Yes | Yes | Yes | Yes | Yes | Yes | Yes | Yes |
| 2. Was the patient’s history clearly described and presented as a timeline? | Yes | Yes | Yes | No | Yes | Yes | Yes | Yes | Yes | Yes |
| 3. Was the current clinical condition of the patient on presentation clearly described? | Yes | Yes | Yes | Yes | Yes | Yes | Yes | Yes | Yes | Yes |
| 4. Were diagnostic tests or assessment methods and the results clearly described? | Yes | Yes | Yes | Yes | Yes | Yes | Yes | Yes | Yes | Yes |
| 5. Was the intervention(s) or treatment procedure(s) clearly described? | NA | Yes | Yes | Yes | Yes | Yes | Yes | Yes | Yes | Yes |
| 6. Was the post-intervention clinical condition clearly described? | NA | Yes | Yes | NO | Yes | Yes | Yes | Yes | Yes | Yes |
| 7. Were adverse events (harms) or unanticipated events identified and described? | NA | Na | NA | NA | Yes | NA | NA | Yes | Yes | NA |
| 8. Does the case report provide takeaway lessons? | Yes | Yes | Yes | Yes | Yes | Yes | Yes | Yes | Yes | Yes |
| **Total score** | 5 | 7 | 7 | 5 | 8 | 7 | 7 | 8 | 8 | 7 |

UC, unclear; NA, not applicable.

# Table S2: JBI Critical Appraisal Checklist for Case series.

| **Checklist** | **Franxman et al. 2014** | **Rao et al. 2015** | **Ng et al. 2019** | **Beaton et al. 2020** | **Strunz et al. 2021** | **Ruiz-Alcaraz et al. 2021** | **Perlman et al. 2021** | **Bucciol et al. 2017** | **Deyà‐Martínez et al. 2018** | **Pac et al. 2020** |
| --- | --- | --- | --- | --- | --- | --- | --- | --- | --- | --- |
| 1. Were there clear criteria for inclusion in the case series? | Yes | Yes | Yes | No | Yes | Yes | No | NO | Yes | Yes |
| 2. Was the condition measured in a standard, reliable way for all participants included in the case series? | Yes | Yes | Yes | Yes | Yes | Yes | Yes | No | Yes | No |
| 3. Were valid methods used for identification of the condition for all participants included in the case series? | Yes | Yes | Yes | Yes | Yes | Yes | Yes | Yes | Yes | Yes |
| 4. Did the case series have consecutive inclusion of participants? | NO | No | No | UC | Yes | Yes | No | Yes | No | Yes |
| 5. Did the case series have complete inclusion of participants? | No | No | No | UC | Yes | Yes | No | No | No | Yes |
| 6. Was there clear reporting of the demographics of the participants in the study? | Yes | Yes | Yes | Yes | Yes | Yes | Yes | Yes | Yes | Yes |
| 7. Was there clear reporting of clinical information of the participants? | Yes | Yes | Yes | Yes | UC | Yes | Yes | Yes | Yes | Yes |
| 8. Were the outcomes or follow up results of cases clearly reported? | Yes | Na | Yes | Yes | Yes | Yes | Yes | Yes | Yes | Yes |
| 9. Was there clear reporting of the presenting site(s)/clinic(s) demographic information? | Yes | Yes | UC | Yes | Yes | Yes | No | NA | Yes | Yes |
| 10. Was statistical analysis appropriate? | Na | Yes | NA | NA | NA | NA | NA | NA | Na | NA |
| **Total score** | 7 | 7 | 6 | 6 | 8 | 9 | 5 | 5 | 7 | 8 |

UC, unclear; NA, not applicable.

# Table S3: Quality of included cohort studies (Newcastle-Ottawa Scale, NOS).

| **Author Year** | **Selection** | | | | **Comparability of cohorts on the basis of the design or analysis** | **Outcome** | | |  | |
| --- | --- | --- | --- | --- | --- | --- | --- | --- | --- | --- |
|  | **Representativeness of the exposed** | **Selection of the non-exposed** | **Ascertainment of exposure** | **Outcome not present at start** |  | **Assessment of outcome** | **follow-up length** | **Adequacy of follow up of** | **Total Quality Score** |  |
| Bates et al. 2004 | 0 | * | * | 0 | * | * | * | * | 6 |  |
| Torigian et al. 2008 | 0 | 0 | * | 0 | * | * | * | * | 5 |  |
| Van de ven et al. 2011 | * | 0 | * | 0 | * | * | * | * | 6 |  |
| Chase et al. 2013 | * | 0 | * | 0 | * | * | * | * | 6 |  |
| Kollert et al. 2014 | 0 | 0 | * | 0 | * | * | 0 | 0 | 3 |  |
| Maglione et al. 2014 | * | 0 | * | 0 | * | * | * | * | 6 |  |
| Maglione et al.2015 | * | 0 | * | 0 | * | * | * | * | 6 |  |
| Maglione et al. 2019 | 0 | 0 | * | 0 | * | * | * | * | 5 |  |
| Verbsky et al. 2020 | * | * | * | 0 | * | * | * | * | 7 |  |
| Fraz et al. 2020 | * | 0 | * | 0 | * | * | 0 | 0 | 4 |  |
| Lopez et al. 2020 | * | 0 | * | 0 | * | * | * | * | 6 |  |
| Meerburg et al. 2020 | 0 | 0 | * | * | * | * | * | * | 6 |  |
| Szczawinska et al. 2021 | 0 | 0 | * | 0 | * | * | * | 0 | 4 |  |
| Lopes et al. 2021 | * | 0 | * | 0 | * | * | * | * | 6 |  |
| Fraz et al. 2022 | * | * | * | 0 | * | * | 0 | 0 | 5 |  |
| Bintalib et al. 2022 | 0 | 0 | * | 0 | * | * | * | * | 5 |  |
| Smits et al. 2022 | 0 | * | * | * | * | * | * | * | 7 |  |

# Table S4: Quality of included case-control studies (NOS).

|  | **Selection** | | | | **Comparability of Cases and Controls on the Basis of the Design or Analysis** | **Outcome** | | |  |
| --- | --- | --- | --- | --- | --- | --- | --- | --- | --- |
| **Author Year** | **Is the Case Definition Adequate?** | **Representativeness of the Cases** | **Selection of Controls** | **Definition of Controls** |  | **Ascertainment of Exposure** | **Same method of ascertainment for cases and controls** | **Non-Response Rate** | **Total Quality Score** |
| Bouvry et al. 2013 | * | * | 0 | * | * | * | * | 0 | 6 |
| Mannina et al. 2016 | * | * | * | * | * | * | * | 0 | 7 |
| Hartono et al. 2017 | * | * | * | * | * | * | * | 0 | 7 |
| Friedmann et al. 2021 | * | * | 0 | 0 | * | * | * | 0 | 5 |

# Table S5: Quality of included cross sectional studies (NOS- adapted for cross sectional studies).

|  | **Selection** | | | | **Comparability of cohorts on the basis of the design or analysis** | **Outcome** | |  |
| --- | --- | --- | --- | --- | --- | --- | --- | --- |
| **Author Year** | **Representativeness of the sample:** | **Sample size** | **Non-respondents** | **Ascertainment of the exposure** |  | **Assessment of outcome** | **Statistical test** | **Total Quality Score** |
| Maarschalk-Ellerbroek et al. 2014 | 0 | 0 | 0 | * | * | ** | * | 5 |
| Hanitsch et al. 2019 | * | 0 | 0 | * | * | ** | 0 | 4 |
| Cinetto et al. 2021 | * | * | 0 | * | * | * | * | 6 |
| Cabanero et al. 2022 | * |  | 0 | * | * | * | * | 5 |

The star rating according to the Newcastle-Ottawa Scale indicate; **Good quality**: 3 or 4 stars in selection domain AND 1 or 2 stars in comparability domain AND 2 or 3 stars in outcome/exposure domain. **Fair quality:** 2 stars in selection domain AND 1 or 2 stars in comparability domain AND 2 or 3 stars in outcome/exposure domain. **Poor quality:** 0 or 1 star in selection domain OR 0 stars in comparability domain OR 0 or 1 stars in outcome/exposure domain.

# Table S6: References for table 2 Diagnostic tests in the work-up of suspected GLILD.

| Tests | Articles |
| --- | --- |
| Radiology |  |
| Chest X-ray | (1-16) |
| CT | (1-11, 16-35) (13, 17, 36-46) |
| PET | (9, 20, 24, 25, 27, 29) (40) (47) |
| Pulmonary function tests | (2, 4-7, 9-14, 17, 18, 20-26, 28-35, 39, 42, 44, 45, 48-55) |
| Bronchoalveolar lavage |  |
| BAL- culture | (1, 2, 5-8, 10, 11, 16, 21, 22, 25, 27, 31, 32, 34, 56) |
| BAL- differential cell count | (4-6, 11, 14, 16, 25, 32, 34, 48, 49, 54-57) |
| Biopsy | (1-11, 16-35) (12, 14, 15, 45, 48-53, 57) |
| TBB | (5-7, 11, 20, 21, 24, 28, 32, 34, 40, 48, 51, 53-55, 57) |
| TBLC | (34) |
| VATS | (4, 5, 10, 16, 23, 24, 26, 29-31, 35, 37, 49, 51-53, 55) |
| Thoracotomy (open surgery) | (1-3, 7, 18, 19, 32, 52, 53) |
| Lung but not specified | (8, 12, 14, 15, 17, 19, 22, 33, 34, 39, 48, 58, 59) |
| Other site | (21, 25, 29, 34, 39, 51, 53) |
| Genetic | (13, 15, 18, 22, 29, 31, 33-35, 38, 40, 49-51, 53-55) |

**References:**

1. Sacco O, Fregonese B, Picco P, Faraci M, Facchetti P, Pistoia V, et al. Common variable immunodeficiency presenting in a girl as lung infiltrates and mediastinal adenopathies leading to severe 'superior vena caval' syndrome. European Respiratory Journal. 1996;9:1958-61.

2. Davies CW, Juniper MC, Gray W, Gleeson FV, Chapel HM, Davies RJ. Lymphoid interstitial pneumonitis associated with common variable hypogammaglobulinaemia treated with cyclosporin A. Thorax. 2000;55(1):88-90.

3. Thatayatikom A, Thatayatikom S, White AJ. Infliximab treatment for severe granulomatous disease in common variable immunodeficiency: a case report and review of the literature. Ann Allergy Asthma Immunol. 2005;95(3):293-300.

4. Matsubara M, Koizumi T, Wakamatsu T, Fujimoto K, Kubo K, Honda T. Lymphoid interstitial pneumonia associated with common variable immunoglobulin deficiency. Intern Med. 2008;47(8):763-7.

5. Boujaoude Z, Arya R, Rafferty W, Dammert P. Organising pneumonia in common variable immunodeficiency. BMJ Case Rep. 2013;2013.

6. Vitale J, Convers KD, Goretzke S, Guzman M, Noyes B, Parkar N, et al. Serum IL-12 and soluble IL-2 receptor levels as possible biomarkers of granulomatous and lymphocytic interstitial lung disease in common variable immunodeficiency: A case report. Journal of Allergy and Clinical Immunology: In Practice. 2015;3:273-6.

7. Tashtoush B, Memarpour R, Ramirez J, Bejarano P, Mehta J. Granulomatous-lymphocytic interstitial lung disease as the first manifestation of common variable immunodeficiency. Clinical Respiratory Journal. 2016;12:337-43.

8. Tessarin G, Bondioni MP, Rossi S, Palumbo L, Soresina A, Badolato R, et al. Rituximab as a Single Agent for Granulomatous Lymphocytic Interstitial Lung Disease in Common Variable Immune Deficiency. J Investig Allergol Clin Immunol. 2019;29(6):470-1.

9. Cowen JE, Stevenson J, Paravasthu M, Darroch J, Jacob A, Tueger S, et al. Common variable immunodeficiency with granulomatous-lymphocytic interstitial lung disease and preceding neurological involvement: A case-report. BMC Pulmonary Medicine. 2020;20.

10. Pattanaik D, Ritter S, Fahhoum J. Common variable immunodeficiency (CVID) with granulomatous interstitial lung disease (GLILD) and SARS COVID-19 infection: case report and review of literature. Allergy, Asthma and Clinical Immunology. 2021;17.

11. Perlman DM, Sudheendra MT, Racilla E, Allen TL, Joshi A, Bhargava M. Granulomatous-Lymphocytic Interstitial Lung Disease Mimicking Sarcoidosis. Sarcoidosis Vasculitis & Diffuse Lung Diseases. 2021;38(3):e2021025.

12. Bates CA, Ellison MC, Lynch DA, Cool CD, Brown KK, Routes JM. Granulomatous-lymphocytic lung disease shortens survival in common variable immunodeficiency. J Allergy Clin Immunol. 2004;114(2):415-21.

13. van de Ven AA, de Jong PA, Hoytema van Konijnenburg DP, Kessels OA, Boes M, Sanders EA, et al. Airway and interstitial lung disease are distinct entities in paediatric common variable immunodeficiency. Clin Exp Immunol. 2011;165(2):235-42.

14. Bouvry D, Mouthon L, Brillet PY, Kambouchner M, Ducroix JP, Cottin V, et al. Granulomatosis-associated common variable immunodeficiency disorder: a case-control study versus sarcoidosis. Eur Respir J. 2013;41(1):115-22.

15. Lopes JP, Ho HE, Cunningham-Rundles C. Interstitial Lung Disease in Common Variable Immunodeficiency. Front Immunol. 2021;12:605945.

16. Hasegawa M, Sakai F, Okabayashi A, Sato A, Yokohori N, Katsura H, et al. Intravenous immunoglobulin monotherapy for granulomatous lymphocytic interstitial lung disease in common variable immunodeficiency. Internal Medicine. 2017;56:2899-902.

17. Torigian DA, LaRosa DF, Levinson AI, Litzky LA, Miller WT, Jr. Granulomatous-lymphocytic interstitial lung disease associated with common variable immunodeficiency: CT findings. J Thorac Imaging. 2008;23(3):162-9.

18. Franxman TJ, Howe LE, Baker JR, Jr. Infliximab for treatment of granulomatous disease in patients with common variable immunodeficiency. J Clin Immunol. 2014;34(7):820-7.

19. Rao N, Mackinnon AC, Routes JM. Granulomatous and lymphocytic interstitial lung disease: a spectrum of pulmonary histopathologic lesions in common variable immunodeficiency--histologic and immunohistochemical analyses of 16 cases. Hum Pathol. 2015;46(9):1306-14.

20. Jolles S, Carne E, Brouns M, El-Shanawany T, Williams P, Marshall C, et al. FDG PET-CT imaging of therapeutic response in granulomatous lymphocytic interstitial lung disease (GLILD) in common variable immunodeficiency (CVID). Clinical and Experimental Immunology. 2016;187:138-45.

21. Pathria M, Urbine D, Zumberg MS, Guarderas J. Management of granulomatous lymphocytic interstitial lung disease in a patient with common variable immune deficiency. BMJ Case Rep. 2016;2016.

22. Bucciol G, Petrone A, Putti MC. Efficacy of mycophenolate on lung disease and autoimmunity in children with immunodeficiency. Pediatr Pulmonol. 2017;52(10):E73-E6.

23. Limsuwat C, Daroca PJ, Lasky JA. A 56-Year-Old-Man With Common Variable Immunodeficiency and Worsening Dyspnea. Chest. 2017;154:e27-e30.

24. Routes JM, Verbsky JW. Immunodeficiency presenting as an undiagnosed disease. Pediatric Clinics. 2017;64(1):27-37.

25. Zdziarski P, Gamian A, Dworacki G. A case report of lymphoid intestitial pneumonia in common variable immunodeficiency: Oligoclonal expansion of effector lymphocytes with preferential cytomegalovirus-specific immune response and lymphoproliferative disease promotion. Medicine (Baltimore). 2017;96(23):e7031.

26. Arraya M, Castro Y, Navarro J, Sarmiento E, Fernández-Cruz E, Carbone J. Rituximab for granulomatous lymphocytic interstitial lung disease in a patient with common variable immunodeficiency. Is single therapy enough. Int J Clin Reumatol. 2018;13(1):38-42.

27. Deyà‐Martínez A, Esteve‐Solé A, Vélez‐Tirado N, Celis V, Costa J, Cols M, et al. Sirolimus as an alternative treatment in patients with granulomatous‐lymphocytic lung disease and humoral immunodeficiency with impaired regulatory T cells. Pediatric Allergy and Immunology. 2018;29(4):425-32.

28. Shah JL, Amin SB, Verma N, Mohammed TL. Granulomatous-Lymphocytic Interstitial Lung Disease in a Patient With Common Variable Immunodeficiency. Current Problems in Diagnostic Radiology. 2018;47:282-4.

29. Sood AK, Funkhouser W, ly B, Weston B, Wu EY. Granulomatous-Lymphocytic Interstitial Lung Disease in 22q11.2 Deletion Syndrome: a Case Report and Literature Review. Current Allergy and Asthma Reports. 2018;18.

30. Ng J, Wright K, Alvarez M, Hunninghake GM, Wesemann DR. Rituximab Monotherapy for Common Variable Immune Deficiency-Associated Granulomatous-Lymphocytic Interstitial Lung Disease. Chest. 2019;155:e117-e21.

31. Tillman R, Guillerman RP, Trojan T, Silva-Carmona M, Chinn IK. Treatment-responsive Granulomatous-Lymphocytic Interstitial Lung disease in a pediatric case of common variable immunodeficiency. Frontiers in Pediatrics. 2019;7.

32. Beaton TJ, Gillis D, Morwood K, Bint M. Granulomatous lymphocytic interstitial lung disease: limiting immunosuppressive therapy-a single-centre experience. Respirology Case Reports. 2020;8.

33. Pac M, Bielecka T, Grzela K, Komarnicka J, Langfort R, Koltan S, et al. Interstitial Lung Disease in Children With Selected Primary Immunodeficiency Disorders-A Multicenter Observational Study. Front Immunol. 2020;11:1950.

34. Ruiz-Alcaraz S, Gaya Garcia-Manso I, Marco-De La Calle FM, Garcia-Mullor MDM, Lopez-Brull H, Garcia-Sevila R. Granulomatous lymphocytic interstitial lung disease: description of a series of 9 cases. Med Clin (Barc). 2021;156(7):344-8.

35. Strunz PP, Frohlich M, Gernert M, Schwaneck EC, Nagler LK, Kroiss A, et al. Rituximab for the treatment of common variable immunodeficiency (CVID) with pulmonary and central nervous system involvement. Open Rheumatology Journal. 2021;15:9-15.

36. Maglione PJ, Overbey JR, Radigan L, Bagiella E, Cunningham-Rundles C. Pulmonary radiologic findings in common variable immunodeficiency: Clinical and immunological correlations. Annals of Allergy, Asthma and Immunology. 2014;113:452-9.

37. Maglione PJ, Overbey JR, Cunningham-Rundles C. Progression of Common Variable Immunodeficiency Interstitial Lung Disease Accompanies Distinct Pulmonary and Laboratory Findings. Journal of Allergy and Clinical Immunology: In Practice. 2015;3:941-50.

38. Maglione PJ, Gyimesi G, Cols M, Radigan L, Ko HM, Weinberger T, et al. BAFF-driven B cell hyperplasia underlies lung disease in common variable immunodeficiency. Jci Insight. 2019;4(5):07.

39. Lopez AL, Paolini MV, Fernandez Romero DS. Lung disease in patients with common variable immunodeficiency. Allergol Immunopathol (Madr). 2020;48(6):720-8.

40. Fraz MSA, Moe N, Revheim ME, Stavrinou ML, Durheim MT, Nordoy I, et al. Granulomatous-Lymphocytic Interstitial Lung Disease in Common Variable Immunodeficiency-Features of CT and <sup>18</sup>F-FDG Positron Emission Tomography/CT in Clinically Progressive Disease. Frontiers in Immunology. 2020;11.

41. Meerburg JJ, Hartmann IJC, Goldacker S, Baumann U, Uhlmann A, Andrinopoulou ER, et al. Analysis of Granulomatous Lymphocytic Interstitial Lung Disease Using Two Scoring Systems for Computed Tomography Scans-A Retrospective Cohort Study. Frontiers in Immunology. 2020;11:589148.

42. von Spee-Mayer C, Echternach C, Agarwal P, Gutenberger S, Soetedjo V, Goldacker S, et al. Abatacept Use Is Associated with Steroid Dose Reduction and Improvement in Fatigue and CD4-Dysregulation in CVID Patients with Interstitial Lung Disease. Journal of Allergy and Clinical Immunology-in Practice. 2021;9(2):760-+.

43. Fraz MSA, Michelsen AE, Moe N, Aaløkken TM, Macpherson ME, Nordøy I, et al. Raised Serum Markers of T Cell Activation and Exhaustion in Granulomatous-Lymphocytic Interstitial Lung Disease in Common Variable Immunodeficiency. Journal of clinical immunology. 2022:1-11.

44. Maarschalk-Ellerbroek LJ, de Jong PA, van Montfrans JM, Lammers JWJ, Bloem AC, Hoepelman AIM, et al. CT Screening for Pulmonary Pathology in Common Variable Immunodeficiency Disorders and the Correlation with Clinical and Immunological Parameters. Journal of Clinical Immunology. 2014;21.

45. Hartono S, Motosue MS, Khan S, Rodriguez V, Iyer VN, Divekar R, et al. Predictors of granulomatous lymphocytic interstitial lung disease in common variable immunodeficiency. Annals of Allergy, Asthma and Immunology. 2017;118:614-20.

46. Smits B, Goldacker S, Seneviratne S, Malphettes M, Longhurst H, Mohamed OE, et al. The efficacy and safety of systemic corticosteroids as first line treatment for granulomatous lymphocytic interstitial lung disease. J Allergy Clin Immunol. 2022.

47. Jolles S, Carne E, Brouns M, El-Shanawany T, Williams P, Marshall C, et al. FDG PET-CT imaging of therapeutic response in granulomatous lymphocytic interstitial lung disease (GLILD) in common variable immunodeficiency (CVID). Clinical and Experimental Immunology. 2017;187(1):138-45.

48. Mannina A, Chung JH, Swigris JJ, Solomon JJ, Huie TJ, Yunt ZX, et al. Clinical Predictors of a Diagnosis of Common Variable Immunodeficiency-related Granulomatous-Lymphocytic Interstitial Lung Disease. Ann Am Thorac Soc. 2016;13(7):1042-9.

49. Cinetto F, Scarpa R, Carrabba M, Firinu D, Lougaris V, Buso H, et al. Granulomatous Lymphocytic Interstitial Lung Disease (GLILD) in Common Variable Immunodeficiency (CVID): A Multicenter Retrospective Study of Patients From Italian PID Referral Centers. Frontiers in Immunology. 2021;12.

50. Cabanero-Navalon MD, Garcia-Bustos V, Forero-Naranjo LF, Baettig-Arriagada EJ, Nunez-Beltran M, Canada-Martinez AJ, et al. Integrating Clinics, Laboratory, and Imaging for the Diagnosis of Common Variable Immunodeficiency-Related Granulomatous-Lymphocytic Interstitial Lung Disease. Frontiers in Immunology. 2022;13.

51. Chase NM, Verbsky JW, Hintermeyer MK, Waukau JK, Tomita-Mitchell A, Casper JT, et al. Use of combination chemotherapy for treatment of granulomatous and lymphocytic interstitial lung disease (GLILD) in patients with common variable immunodeficiency (CVID). Journal of Clinical Immunology. 2013;33:30-9.

52. Szczawinska-Poplonyk A, Jonczyk-Potoczna K, Mikos M, Ossowska L, Langfort R. Granulomatous Lymphocytic Interstitial Lung Disease in a Spectrum of Pediatric Primary Immunodeficiencies. Pediatric and Developmental Pathology. 2021;24:504-12.

53. Verbsky JW, Hintermeyer MK, Simpson PM, Feng M, Barbeau J, Rao N, et al. Rituximab and antimetabolite treatment of granulomatous and lymphocytic interstitial lung disease in common variable immunodeficiency. Journal of Allergy and Clinical Immunology. 2020;147:704-12.e17.

54. Hanitsch LG, Wittke K, Stittrich AB, Volk HD, Scheibenbogen C. Interstitial Lung Disease Frequently Precedes CVID Diagnosis. J Clin Immunol. 2019;39(8):849-51.

55. Nishimura M, Miyata J, Tanigaki T, Nomura S, Serizawa Y, Igarashi S, et al. Successful Treatment of Granulomatous-lymphocytic Interstitial Lung Disease in a Patient with CTLA-4 Deficiency. Intern Med. 2022.

56. Friedmann D, Unger S, Keller B, Rakhmanov M, Goldacker S, Zissel G, et al. Bronchoalveolar Lavage Fluid Reflects a T<inf>H</inf>1-CD21<sup>low</sup> B-Cell Interaction in CVID-Related Interstitial Lung Disease. Frontiers in Immunology. 2021;11.

57. Kollert F, Venhoff N, Goldacker S, Wehr C, Lutzen N, Voll RE, et al. Bronchoalveolar lavage cytology resembles sarcoidosis in a subgroup of granulomatous CVID. European Respiratory Journal.43(3):922-4.

58. Huston J, Johnson J, Hemnes A, Pugh M. Evidence of pulmonary arterial hypertension in two patients with common variable immunodeficiency. Pulm Circ. 2020;10(2):2045894020922792.

59. Maglione PJ, Ko HBM, Beasley MB, Strauchen JA, Cunningham-Rundles C. Tertiary lymphoid neogenesis is a component of pulmonary lymphoid hyperplasia in patients with common variable immunodeficiency. Journal of Allergy and Clinical Immunology. 2014;133(2):535-42.
